# Supplementary material for: Significance of the drug-induced lymphocyte stimulation test for various oral mesalamines in ulcerative colitis with mesalamine intolerance
Source: PLoS One. 2025 Oct 30;20(10):e0334969. doi: 10.1371/journal.pone.0334969 (PMC12574853; doi:10.1371/journal.pone.0334969)
Supplement: Attachment — Submitted filename: Response to Reviewers.docx [file pone.0334969.s001.docx]

Manuscript ID: PONE-D-25-17000

Title: Significance of the drug-induced lymphocyte stimulation test for various oral mesalamines in ulcerative colitis with mesalamine intolerance

Dear Editors and Reviewers,

We would like to thank you for the opportunity to revise our manuscript and the insightful comments. We have carefully addressed each point raised by the reviewers and have revised the manuscript accordingly. Our detailed responses to each comment are provided below.

Reviewer #1

Comment: The clinical usefulness and reliability of DLST testing for mesalazine is very low. For this reason, DLST testing is being performed less and less. As a result, it is difficult to find meaning in research discussing the usefulness of DLST testing. There have already been several papers reporting that mesalazine intolerance has a poor prognosis, so this study lacks novelty.

Response:

Given its relatively low sensitivity and specificity, the use of DLST in clinical practice is limited. Further, its application in other settings has decreased. However, in Japan, DLST is still used as a supportive tool for evaluating suspected drug-induced hypersensitivity reactions, such as mesalamine intolerance.

This study differs from previous ones because previous studies commonly assessed the DLST results only for mesalamine preparation that were initially suspected. Moreover, the present study examined the DLST results for various types of oral mesalamine in each patient, thereby allowing the evaluation of whether a higher M-DLST across various formulations is associated with clinical intolerance and retreatment failure.

In addition, this study explored an optimal SI cutoff value for predicting intolerance, which has not been clearly addressed in previous studies. We believe that our findings offer novel contributions to the field, particularly in using DLST more effectively as part of a multifaceted diagnostic approach. These points have been added to the Introduction and Discussion of the revised manuscript.

Comment: The number of cases is small.

Response:

We acknowledge that this single-center retrospective study was limited by the small sample size. This has been explicitly discussed in the revised Discussion.

Reviewer #2

Comment 1: The limited sample size (n=28) may result in insufficient statistical power. Expanding the cohort or conducting multicenter collaborations would improve the reliability of the findings.

Response:

We agree with this limitation, and this has been clearly stated in the Discussion. We also recommended the need to conduct multicenter prospective studies to validate our findings.

Comment 2: The exclusion of certain cases (e.g., patients receiving topical mesalazine) during screening could introduce selection bias. The potential impact of this limitation should be explicitly addressed in the Discussion section.

Response:

Thank you for this valuable suggestion. A statement acknowledging the possibility of selection bias due to our exclusion criteria and its potential effect on the generalizability of the results has been added to the Discussion.

Comment 3: The manuscript lacks detailed methodological descriptions of the DLST, including drug concentrations and incubation periods. Supplementary procedural details are recommended.

Response:

The Methods was revised to include data on drug concentrations, incubation conditions, and control settings used in DLST.

Comment 4: The immunological mechanisms of DLST positivity and mesalamine intolerance are not discussed in depth (e.g., whether it is an allergic reaction or non-immunotoxic).

Response:

Thank you for your valuable comment. As suggested, the Discussion has been revised. In particular, data on the possible immunological mechanisms underlying mesalamine intolerance and DLST positivity were added. Moreover, information on immune-mediated reactions to 5-ASA, sulfapyridine, or formulation excipients, a genome-wide significant SNP (rs144384547) associated with mesalamine-induced symptoms, and possible involvement of dysbiosis was included. Furthermore, an explanation of the DLST principle was added, thereby emphasizing its relevance in detecting T-cell-mediated hypersensitivity, which may be reflected by a high stimulation index in mesalamine-intolerant cases.

Comment 5: The conclusions emphasise the predictive value of DLST, but there may be a problem of false positives/negatives in practical application. Discussion is needed on how to improve diagnostic accuracy.

Response:

The Conclusion and Discussion were revised to support our claims regarding predictive utility. The risks of false-positive and false-negative results were explicitly discussed. Furthermore, we proposed interpreting the DLST findings in consideration of clinical symptoms and, if available, genetic or immunologic markers.

Additional Revisions

We appreciate the editorial request regarding data sharing. As described in the manuscript, the data were anonymized before analysis in compliance with ethical guidelines and were collected using an opt-out procedure approved by our institutional ethics committee. The requirement for informed consent was waived owing to the retrospective nature of the study and the use of anonymized data.

However, after re-examining the dataset in light of journal publication standards, certain combinations of clinical variables (e.g., age, sex, disease characteristics, and treatment responses) could potentially allow the reidentification of individuals if shared externally. Furthermore, participants did not provide explicit consent for external data sharing. According to Japanese law, disclosing potentially identifiable clinical information to parties outside the institution without consent may constitute a legal violation. Therefore, we have removed the Supporting Information and clarified this limitation in the revised Data availability section.

The revised manuscript with track changes, a clean version of the manuscript, and a response letter were uploaded separately.

We confirmed that our protocols do not require registration in protocols.io, as they do not involve novel laboratory methods.

We hope that the revisions and clarifications satisfactorily address the concerns raised. We greatly appreciate the reviewers’ constructive feedback and your consideration of our revised manuscript.

Sincerely,

Akira Madarame, MD

On behalf of all co-authors
