# Supplementary material for: Significance of the drug-induced lymphocyte stimulation test for various oral mesalamines in ulcerative colitis with mesalamine intolerance
Source: PLoS One. 2025 Oct 30;20(10):e0334969. doi: 10.1371/journal.pone.0334969 (PMC12574853; doi:10.1371/journal.pone.0334969)
Supplement: Attachment — Submitted filename: review-PONE.docx [file pone.0334969.s002.docx]

The manuscript addresses a clinically relevant question—whether DLST results across multiple oral 5-ASA formulations can help diagnose mesalamine intolerance and predict retreatment outcomes. While the topic is of interest, several methodological issues substantially limit the robustness and generalizability of the conclusions. Although both F-DLST and M-DLST showed statistically significant associations with mesalamine intolerance, their limited sensitivity and specificity substantially restrict their clinical utility.

**Major comments**

**Materials and methods**

Why were patients excluded with previous topical 5-ASA drugs? It is a potential confounder, and excluding them results in selection bias.

It should be emphasized that reintroduction of a different type of 5-ASA formulation is only acceptable and ethical in cases of mild intolerance. In cases of severe adverse events, such as severe renal failure or pancreatitis, re-exposure should be strictly avoided. This important distinction should be clearly stated.

**Results section**

Very small and highly imbalanced sample. The comparison between only six and twenty-two patients—set against an already limited total sample size—substantially restricts statistical power and calls into question the external validity and generalizability of the study’s conclusions.

Insufficient statistical modelling. Only univariate comparisons were performed; potential confounders (age, disease extent, 5-ASA dose, AE severity, corticosteroid use) were not adjusted for. A multivariable logistic regression is essential to clarify independent predictors, however, due to the small sample size, it may not be possible.

Why was 5-ASA reinducted in case of myelosuppression? It should be stated, as current ECCO guidelines advise **against** 5-ASA re-challenge after serious organ toxicity. Was the pancreatitis mild?

Although both F-DLST and M-DLST showed statistically significant associations with mesalamine intolerance, their limited sensitivity and specificity substantially restrict their clinical utility.

The ROC-derived cut-offs yield apparently excellent AUC values; however, because they were optimised and evaluated on the same, very small dataset (n = 28, with only six tolerant cases), the results are likely over-optimistic and may not generalise. External validation in a larger cohort is essential before these thresholds can be recommended for clinical use.

**Minor comments**

**Introduction**

The phrase ‘Salazosulfapyridine (SASP), one of the first 5-ASAs’ is misleading. SASP is a sulfonamide-based prodrug that delivers 5-ASA after bacterial azo-reduction in the colon. Because of its sulfapyridine moiety, SASP differs chemically, pharmacokinetically, and in its adverse-event profile from the later, pure mesalazine formulations. Please rephrase it.

**Matherials and methods**

As Salazopyrin is not a classical 5-ASA and , I would not include it in the study as it can increase bias. Both its pharmacokinetics and immunologic toxicity differ substantially from those of single-agent 5-ASA preparations. However, as I can see it in the methods section, no patients received Salazopyrin among the observed 28 patients.
