# Supplementary material for: Significance of the drug-induced lymphocyte stimulation test for various oral mesalamines in ulcerative colitis with mesalamine intolerance
Source: PLoS One. 2025 Oct 30;20(10):e0334969. doi: 10.1371/journal.pone.0334969 (PMC12574853; doi:10.1371/journal.pone.0334969)
Supplement: Attachment — Submitted filename: Response_to_Reviewers_auresp_2.docx [file pone.0334969.s003.docx]

Manuscript ID: PONE-D-25-17000

Title: Significance of the drug-induced lymphocyte stimulation test for various oral mesalamines in ulcerative colitis with mesalamine intolerance

Dear Editors and Reviewers:

We would like to thank you for the opportunity to revise our manuscript as well as for the insightful comments. We have carefully addressed each point raised by the reviewers and have revised the manuscript accordingly. Our detailed responses to each comment are provided below.

Reviewer #3

Comment 1: The authors divided the 28 patients who experienced AEs from the first 5-ASA treatment into two groups: a tolerable group of six and an intolerable group of 22. Was this determined by retreatment after discontinuing the first 5-ASA to assess tolerability or intolerance (Page 6, line 107-111)? The Results section states 14 patients underwent retreatment. The study flow and Methods section needs to be clearly described.

Response:

Thank you for your valuable comment. We apologize for the lack of clarity in the description regarding the patient grouping. In the revised manuscript, we have clarified that mesalamine intolerance was defined as either (1) the occurrence of adverse events (AEs) within 6 months of the first oral 5-ASA and the inability to continue taking oral mesalamines owing to intolerance upon retreatment or (2) patients whose initial AEs were severe or involved significant organ toxicity, and therefore, did not receive mesalamine retreatment. We have added these definitions and clarifications to the Methods section, under the heading Data collection and definitions (Page 6, lines 119–124).

Comment 2: The definitions of success and failure for 5-ASA retreatment should be indicated. Fig 2 shows that mesalamine retreatment was undergone multiple times. However, it is unclear at what point the retreatment was deemed a success or failure.

Response:

Thank you for bringing this to our attention. We have added clear definitions of “retreatment success” and “failure” to the Methods section, under the heading Data collection and definitions. Retreatment success was defined as the ability to continue taking at least one oral mesalamine formulation for >6 months after starting the first oral 5-ASA without AEs, and retreatment failure was defined as discontinuation of mesalamine retreatment owing to AEs (Page 6, lines 126–129).

To make this clearer, we have revised the description of Fig. 2 as well.

Comment 3: The authors should provide information on mesalamine retreatment details that could affect outcomes, such as dosage and the presence or absence of desensitization therapy.

Response:

Thank you for your insightful comment. We have added detailed information in the Results section on the methods of mesalamine retreatment that could affect outcomes. Specifically, we describe the three approaches used, which are as follows: (1) switching to a different type of oral 5-ASA, (2) performing desensitization therapy, and (3) switching to SASP.

Additionally, the initial dose at retreatment was specified, and all treatment regimens were titrated upward to reach at least 2,000 mg/day (Page 14, lines 248–261).

Comment 4: The spelling of mesalamine should be consistent throughout the text and figures.

Response:

Thank you for bringing this to our attention. Accordingly, we have carefully checked the entire manuscript, including the text and figures, to ensure that the spelling of mesalamine is consistent throughout.

Major comments

Materials and methods

Comment 1: Why were patients excluded with previous topical 5-ASA drugs? It is a potential confounder, and excluding them results in selection bias.

Response: Thank you for your insightful comment. We acknowledge the reports regarding mesalamine intolerance caused by topical 5-ASA drugs. However, this study specifically aimed to investigate mesalamine intolerance and retreatment outcomes in patients who experienced AEs from their first dose of oral 5-ASA formulation. The inclusion of patients who only experienced intolerance to topical 5-ASA preparations may introduce heterogeneity as their pharmacokinetics and routes of exposure are different from those of oral formulations. Therefore, patients who previously used topical 5-ASA were excluded to maintain the consistency and validity of the study cohort. For clarity, we have added this rationale to the Materials and Methods section (Page 5, lines 102–108).

Comment 2: It should be emphasized that reintroduction of a different type of 5-ASA formulation is only acceptable and ethical in cases of mild intolerance. In cases of severe adverse events, such as severe renal failure or pancreatitis, re-exposure should be strictly avoided. This important distinction should be clearly stated.

Response: Thank you for this important comment. We agree that retreatment with a different type of 5-ASA formulation should only be considered in cases of mild intolerance without major organ damage.

To clarify this ethical consideration, we have added the following sentence to the Materials and Methods section: “Patients who experienced only mild AEs from the first oral 5-ASA and had no organ damage received mesalamine retreatment after consultation with their attending physician.” (Page 6, lines 124–126)

Results section

Comment 1: Very small and highly imbalanced sample. The comparison between only six and twenty-two patients—set against an already limited total sample size—substantially restricts statistical power and calls into question the external validity and generalizability of the study’s conclusions.

Insufficient statistical modelling. Only univariate comparisons were performed; potential confounders (age, disease extent, 5-ASA dose, AE severity, corticosteroid use) were not adjusted for. A multivariable logistic regression is essential to clarify independent predictors, however, due to the small sample size, it may not be possible.

Response: Thank you for pointing out this important limitation. We completely agree that the small and uneven sample size restricts the statistical power and limits the external validity and generalizability of our study findings.

In addition, only univariate analyses were performed, and adjusting for potential confounders such as age, disease extent, 5-ASA dose, AE severity, and corticosteroid use would have strengthened the conclusions. However, considering the limited number of patients enrolled in this retrospective single-center cohort, conducting a robust multivariable logistic regression analysis was not feasible.

To address this concern, we have clearly discussed these limitations in the Discussion section and have emphasized that future studies should validate our findings in larger, multicenter prospective cohorts, with adequate sample sizes, to allow for multivariable adjustment (Page 21–22, lines 396–416).

Comment 2: Why was 5-ASA reinducted in case of myelosuppression? It should be stated, as current ECCO guidelines advise against 5-ASA re-challenge after serious organ toxicity. Was the pancreatitis mild?

Response: Thank you for raising this important point. We agree that mesalamine reintroduction should be strictly avoided in patients with significant organ toxicity, such as myelosuppression or severe pancreatitis, in line with current ECCO guidelines.

In this study, no patients with serious organ toxicity received mesalamine retreatment. We believe that this misunderstanding may have arisen from the original wording of our definition of mesalamine intolerance.

To clarify this ethical consideration, we have revised the definition in the Materials and Methods section to explicitly state that retreatment was only performed in cases without significant organ damage (Page 6, lines 124–126).

Comment 3: Although both F-DLST and M-DLST showed statistically significant associations with mesalamine intolerance, their limited sensitivity and specificity substantially restrict their clinical utility.

The ROC-derived cut-offs yield apparently excellent AUC values; however, because they were optimised and evaluated on the same, very small dataset (n = 28, with only six tolerant cases), the results are likely over-optimistic and may not generalise. External validation in a larger cohort is essential before these thresholds can be recommended for clinical use.

Response: Thank you for this valuable comment. We agree that despite the significant associations of F-DLST and M-DLST with mesalamine intolerance, their limited sensitivity and specificity substantially restrict their current clinical utility.

We also acknowledge that the ROC-derived cutoff values and AUCs may appear overly optimistic because they were derived and evaluated within the same small cohort (n = 28), which included only six tolerant cases.

To address this, we have further discussed these limitations in the Discussion section and have emphasized that external validation using larger, multicenter prospective cohorts is essential before these cutoff values can be recommended for routine clinical practice (Page 22, lines 405–410).

Minor comments

Introduction

Comment 1: The phrase ‘Salazosulfapyridine (SASP), one of the first 5-ASAs’ is misleading. SASP is a sulfonamide-based prodrug that delivers 5-ASA after bacterial azo-reduction in the colon. Because of its sulfapyridine moiety, SASP differs chemically, pharmacokinetically, and in its adverse-event profile from the later, pure mesalazine formulations. Please rephrase it.

Response: Thank you for raising this point. We agree that the original phrasing may have been misleading. As suggested, we have revised the sentence to clarify that SASP is a sulfonamide-based prodrug that delivers 5-ASA to the colon via bacterial azo-reduction and that it differs chemically, pharmacokinetically, and in its adverse event profile from the later, pure mesalamine formulations (Page 3, lines 45–49).

Materials and methods

Comment 1: As Salazopyrin is not a classical 5-ASA and , I would not include it in the study as it can increase bias. Both its pharmacokinetics and immunologic toxicity differ substantially from those of single-agent 5-ASA preparations. However, as I can see it in the methods section, no patients received Salazopyrin among the observed 28 patients.

Response: Thank you for your comment. We agree that the inclusion of patients who received SASP could introduce bias because SASP has substantially different pharmacokinetics and immunologic toxicity from single-agent 5-ASA formulations.
To avoid this potential bias, we have explicitly stated in the Materials and Methods that patients who initiated oral SASP at diagnosis were excluded from the study (Page 5, line 99–100).

The revised manuscript with tracked changes, a clean version of the manuscript, and a response letter were uploaded separately.

We confirmed that our protocols do not require registration in protocols.io because they do not involve novel laboratory methods.

We hope that the revisions and clarifications satisfactorily address the concerns of the reviewers. We greatly appreciate the constructive feedback of the reviewers and your consideration of our revised manuscript. We hope that the revised manuscript will be acceptable for publication in *PLOS ONE*.

Sincerely,

Akira Madarame, MD

On behalf of all co-authors
