# Supplementary material for: Significance of the drug-induced lymphocyte stimulation test for various oral mesalamines in ulcerative colitis with mesalamine intolerance
Source: PLoS One. 2025 Oct 30;20(10):e0334969. doi: 10.1371/journal.pone.0334969 (PMC12574853; doi:10.1371/journal.pone.0334969)
Supplement: Attachment — Submitted filename: Response_to_Reviewers_auresp_3.docx [file pone.0334969.s004.docx]

Manuscript ID: PONE-D-25-17000R2

Title: Significance of the drug-induced lymphocyte stimulation test for various oral mesalamines in ulcerative colitis with mesalamine intolerance

Dear Editors and Reviewers:

We sincerely thank the Academic Editor and Reviewers for their thoughtful and constructive comments, which have been very helpful in improving our manuscript. We have carefully revised the manuscript in accordance with the suggestions, and the specific responses to each comment are detailed below. Page and line numbers refer to the revised version of the manuscript.

Reviewer #3

Comment: The authors have responded properly to the reviewer's comments, so I have no additional comments.

Response:

We thank the reviewer for the positive evaluation and for acknowledging our previous revisions. We are grateful for the constructive feedback provided during the review process.

Reviewer #5

Comment (Statistical limitations):

The study is based on a small, single-center cohort (n=28), which limits statistical power. The cutoff values derived from ROC analysis and the odds ratios may be subject to overfitting. The authors should acknowledge this more explicitly and emphasize the need for validation in larger, multicenter prospective studies.

Response:

We thank the reviewer for raising this important point. We fully agree that the small, single-center cohort limits the statistical power and may increase the risk of overfitting for ROC-derived cutoff values and odds ratios. In the revised Discussion, we have explicitly acknowledged these limitations and emphasized the need for validation in larger, multicenter prospective studies to confirm the diagnostic performance and clinical applicability of DLST (page 22, lines 413–417).

Comment (Clinical applicability of DLST):

While the results suggest that DLST could be useful in predicting intolerance and retreatment outcomes, its clinical application remains unclear due to potential false positives and negatives. The discussion would be strengthened by presenting specific clinical scenarios (e.g., when DLST should be considered in practice, or how to act upon multiple positive results) and by proposing a preliminary decision-making algorithm.

Response:

We thank the reviewer for this constructive suggestion. In the revised Discussion, we have added specific descriptions of how DLST may be applied in clinical practice. Based on our findings, we suggest that DLST should be performed after the first onset of intolerance symptoms to oral 5-ASA and ideally include all available mesalamine formulations. We further describe a preliminary framework in which retreatment should be avoided when two or more DLSTs are positive, whereas cautious reintroduction may be considered in patients with negative or single-positive results (page 21, lines 388–396). We have also emphasized that this framework is preliminary and requires validation in larger, multicenter prospective studies before being implemented in routine clinical practice (page 22, lines 410–413).

Comment (Adjustment for confounding factors):

The analysis relies only on univariate comparisons. Potential confounders, such as age, type of first 5-ASA, severity of adverse events, and corticosteroid use, may influence the results. Although the limited sample size may preclude multivariable analysis, the authors should discuss this limitation and outline how future research could address it.

Response:

We thank the reviewer for highlighting this important issue. As suggested, we have added a statement in the Discussion acknowledging that our analysis was limited to univariate comparisons and did not adjust for potential confounders, including age, type of first 5-ASA, severity of adverse events, and corticosteroid use. We have also noted that, although multivariable analysis was not feasible due to the small sample size, future studies with larger cohorts will be necessary to incorporate these factors and clarify independent predictors of mesalamine intolerance and retreatment outcomes (page 21-22, lines 397–405).

We carefully reviewed the reviewer comments and confirm that no additional references were specifically recommended in this revision round. Accordingly, no new citations were added.

The revised manuscript with tracked changes, a clean version of the manuscript, and a response letter were uploaded separately.

We confirmed that our protocols do not require registration in protocols.io because they do not involve novel laboratory methods.

We hope that the revisions and clarifications satisfactorily address the concerns of the reviewers. We greatly appreciate the constructive feedback of the reviewers and your consideration of our revised manuscript. We hope that the revised manuscript will be acceptable for publication in *PLOS ONE*.

Sincerely,

Akira Madarame, MD

On behalf of all co-authors
