# Supplementary material for: Significance of the drug-induced lymphocyte stimulation test for various oral mesalamines in ulcerative colitis with mesalamine intolerance
Source: PLoS One. 2025 Oct 30;20(10):e0334969. doi: 10.1371/journal.pone.0334969 (PMC12574853; doi:10.1371/journal.pone.0334969)
Supplement: Attachment — Submitted filename: Response_to_Reviewers_auresp_4.docx [file pone.0334969.s005.docx]

Manuscript ID: PONE-D-25-17000R3

Title: Significance of the drug-induced lymphocyte stimulation test for various oral mesalamines in ulcerative colitis with mesalamine intolerance

Dear Editors and Reviewers:

We sincerely thank the Academic Editor and Reviewers for their thoughtful and constructive comments, which have been very helpful in improving our manuscript. We have carefully revised the manuscript in accordance with the suggestions, and the specific responses to each comment are detailed below. Page and line numbers refer to the revised version of the manuscript.

Reviewer # 6, Comment 1:

According to the Methods, retreatment success was defined as the ability to continue oral mesalamine for more than 6 months. However, in the Results and Fig. 2, some patients classified into the intolerant group appear to have achieved ‘success’ with second- or third-line mesalamine therapy. The subsequent clinical course of these patients (e.g., whether they later discontinued mesalamine due to AEs) is not clearly described. Please clarify how such cases were classified into the final tolerant vs. intolerant groups.

Response:

We sincerely thank the reviewer for this helpful comment. To address the concern:

1. We revised the Results sentence for clarity. The original wording “Fourteen patients underwent retreatment with oral mesalamines after experiencing AEs in response to the first 5-ASA (Fig 2), including six patients who were tolerant to oral mesalamines and eight patients who were intolerant to oral mesalamines” has been replaced with “Fourteen patients underwent retreatment with oral mesalamines after AEs to the first 5-ASA (Fig 2), of whom six achieved retreatment success and were classified into the mesalamine-tolerant group, whereas eight experienced retreatment failure and were classified into the mesalamine-intolerant group.” (page 13, lines 245–248)
2. We added details of the clinical course for patients with successful retreatment: “Six patients who achieved successful retreatment maintained oral mesalamine therapy at a minimum dose of 2,000 mg/day for at least 6 months, and none discontinued treatment thereafter.” (page 14-15, lines 270–273)
3. To avoid ambiguity, we also added a statement in the Methods explicitly clarifying that patients with successful retreatment were classified into the mesalamine-tolerant group, whereas those with retreatment failure were classified into the mesalamine-intolerant group. (page 6, lines 129–131)

We believe these revisions clearly address the reviewer’s concern and eliminate any potential misunderstanding regarding the classification of patients.

**Comment 2:**

In Results (line 185) and Figure 2, the phrase “28 patients with AEs” is mentioned. Should this actually refer to the 28 patients who underwent DLST?

**Response:**

We thank the reviewer for this careful observation. The phrase “28 patients with AEs” was indeed misleading. What we intended to describe was the 28 patients who underwent DLST. We have revised the Results section (page 9, line 186) and the corresponding label in Figure 2 to read “Patients who underwent DLST” to avoid confusion.

**Comment 3:**
In the sentence “All 13 patients with positive F-DLSTs were mesalamine intolerant (p = 0.018, OR 2.444, 95% CI 1.479–4.039) (Table 3),” it would be clearer to state “mesalamine intolerant group” rather than simply “mesalamine intolerant.”

**Response:**
We appreciate the reviewer’s helpful suggestion. We agree that “mesalamine-intolerant group” is clearer and avoids potential ambiguity. We have revised the sentence in the Results section (page 12, line 219) to read: “All 13 patients with positive F-DLSTs were in the mesalamine-intolerant group (p = 0.018, OR 2.444, 95% CI 1.479–4.039) (Table 3).”

**Comment 4:**

There is an inconsistency between the study period reported in the Materials and Methods section and that in the Results section. Please revise accordingly.

**Response:**

We thank the reviewer for pointing out this inconsistency. We have corrected the discrepancy by unifying the study period throughout the manuscript. The study period is now consistently reported as between 2014 and 2021 in both the Materials and Methods and Results sections. (page 9, line 180)

Reviewer #7:

This research paper examines 5-ASA intolerance, a topic that has gained attention in recent years. The content is intriguing. However, as Reviewer 5 noted, unfortunately, the number of patients examined in this study is small. Also, were there no patients among the 22 with mesalamine intolerance who used SASP? Unless the authors evaluated the efficacy of SASP, these patients cannot be considered actual cases of mesalamine intolerance. Please reconsider.

Response:

We thank the reviewer for these constructive comments.

1. We fully acknowledge the limitation of the relatively small sample size. As noted by Reviewer 5, we have already addressed this point in the Limitations section, emphasizing the need for confirmation in larger, multicenter prospective cohorts.
2. With regard to SASP, we confirm that SASP retreatment was performed and evaluated in this study. In fact, SASP was included among the successful retreatment cases. Specifically, two patients who failed initial 5-ASA therapy subsequently tolerated SASP and maintained treatment without recurrence of adverse events. These patients were therefore classified into the mesalamine-tolerant group. Conversely, three patients experienced retreatment failure with SASP (one in the second-line therapy and two in the third-line therapy). We have revised the Results section (page 14, lines 255–259, 262–265) to make these details explicit, thereby clarifying that the efficacy of SASP was fully assessed in our cohort.

We believe these revisions adequately address the reviewer’s concern.

We carefully reviewed the reviewer comments and confirm that no additional references were specifically recommended in this revision round. Accordingly, no new citations were added.

The revised manuscript with tracked changes, a clean version of the manuscript, and a response letter were uploaded separately.

We confirmed that our protocols do not require registration in protocols.io because they do not involve novel laboratory methods.

We hope that the revisions and clarifications satisfactorily address the concerns of the reviewers. We greatly appreciate the constructive feedback of the reviewers and your consideration of our revised manuscript. We hope that the revised manuscript will be acceptable for publication in PLOS ONE.

Sincerely,

Akira Madarame, MD

On behalf of all co-authors
